# Supplementary material for: Hybrid Models and Biological Model Reduction with PyDSTool
Source: PLoS Comput Biol. 2012 Aug 9;8(8):e1002628. doi: 10.1371/journal.pcbi.1002628 (PMC3415397; doi:10.1371/journal.pcbi.1002628)
Supplement: Text S4 — Complete source code for the PyDSTool package (version 0.88.120504). Includes API documentation and help files linking to web pages. This file is identical to the current public release on Sourceforge.net. (ZIP) [file pcbi.1002628.s004.zip › PyDSTool/html/PyDSTool.Toolbox.DSSRT_tools-module.html]

xml version="1.0" encoding="ascii"?


PyDSTool.Toolbox.DSSRT\_tools


| Home | Trees | Indices | Help | PyDSTool |
| --- | --- | --- | --- | --- |

Package PyDSTool ::
Package Toolbox ::
Module DSSRT\_tools

[hide private]

[frames] | no frames]

# Module DSSRT\_tools

source code

```
DSSRT interface tools.
(strongly biased towards neural models at this time).

These utilities do not fully construct the graphical
specification for DSSRT, which still has to be done by hand.
Partial templates for the graphical specs are created.

   Robert Clewley, October 2005.
```


|  |  |
| --- | --- |
| Classes | [hide private] |

DSSRT\_info


|  |  |
| --- | --- |
| Functions | [hide private] |

|  |  |
| --- | --- |
| plotNetworkGraph(dssrt\_obj) | source code |


|  |  |
| --- | --- |
| Variables | [hide private] |

CFGorder = `['VARSEXT', 'VARSINT', 'INPUTS', 'DEQNS', 'CFAC', '...`

nodes\_order = `['NODE', 'NODELABEL', 'NODEADDSTATES', 'NODESTAT...`

ALLOW\_THREADS = `1`

Abs = `Abs (ModelSpec wrapper)`

Acos = `Acos (ModelSpec wrapper)`

Asin = `Asin (ModelSpec wrapper)`

Atan = `Atan (ModelSpec wrapper)`

Atan2 = `Atan2 (ModelSpec wrapper)`

BUFSIZE = `10000`

Betavariate = `Betavariate (ModelSpec wrapper)`

CLIP = `0`

Ceil = `Ceil (ModelSpec wrapper)`

Choice = `Choice (ModelSpec wrapper)`

Cos = `Cos (ModelSpec wrapper)`

Cosh = `Cosh (ModelSpec wrapper)`

Degrees = `Degrees (ModelSpec wrapper)`

E = `QuantSpec e (ExpFuncSpec)`

ERR\_CALL = `3`

ERR\_DEFAULT = `0`

ERR\_DEFAULT2 = `2084`

ERR\_IGNORE = `0`

ERR\_LOG = `5`

ERR\_PRINT = `4`

ERR\_RAISE = `2`

ERR\_WARN = `1`

Exp = `Exp (ModelSpec wrapper)`

Expovariate = `Expovariate (ModelSpec wrapper)`

FLOATING\_POINT\_SUPPORT = `1`

FPE\_DIVIDEBYZERO = `1`

FPE\_INVALID = `8`

FPE\_OVERFLOW = `2`

FPE\_UNDERFLOW = `4`

Fabs = `Fabs (ModelSpec wrapper)`

False\_ = `False`

Floor = `Floor (ModelSpec wrapper)`

Fmod = `Fmod (ModelSpec wrapper)`

Frexp = `Frexp (ModelSpec wrapper)`

Gammavariate = `Gammavariate (ModelSpec wrapper)`

Gauss = `Gauss (ModelSpec wrapper)`

Getrandbits = `Getrandbits (ModelSpec wrapper)`

Getstate = `Getstate (ModelSpec wrapper)`

Hypot = `Hypot (ModelSpec wrapper)`

Infinity = `inf`

Jumpahead = `Jumpahead (ModelSpec wrapper)`

Ldexp = `Ldexp (ModelSpec wrapper)`

Log = `Log (ModelSpec wrapper)`

Log10 = `Log10 (ModelSpec wrapper)`

Lognormvariate = `Lognormvariate (ModelSpec wrapper)`

MAXDIMS = `32`

Max = `Max (ModelSpec wrapper)`

Min = `Min (ModelSpec wrapper)`

Modf = `Modf (ModelSpec wrapper)`

NAN = `nan`

NINF = `-inf`

NZERO = `-0.0`

Normalvariate = `Normalvariate (ModelSpec wrapper)`

PINF = `inf`

PZERO = `0.0`

Paretovariate = `Paretovariate (ModelSpec wrapper)`

Pi = `QuantSpec pi (ExpFuncSpec)`

Pow = `Pow (ModelSpec wrapper)`

RAISE = `2`

Radians = `Radians (ModelSpec wrapper)`

Randint = `Randint (ModelSpec wrapper)`

Random = `Random (ModelSpec wrapper)`

Randrange = `Randrange (ModelSpec wrapper)`

SHIFT\_DIVIDEBYZERO = `0`

SHIFT\_INVALID = `9`

SHIFT\_OVERFLOW = `3`

SHIFT\_UNDERFLOW = `6`

Sample = `Sample (ModelSpec wrapper)`

ScalarType = `(<type 'int'>, <type 'float'>, <type 'complex'>, ...`

Seed = `Seed (ModelSpec wrapper)`

Setstate = `Setstate (ModelSpec wrapper)`

Shuffle = `Shuffle (ModelSpec wrapper)`

Sin = `Sin (ModelSpec wrapper)`

Sinh = `Sinh (ModelSpec wrapper)`

Sqrt = `Sqrt (ModelSpec wrapper)`

Sum = `Sum (ModelSpec wrapper)`

Systemrandom = `Systemrandom (ModelSpec wrapper)`

Tan = `Tan (ModelSpec wrapper)`

Tanh = `Tanh (ModelSpec wrapper)`

True\_ = `True`

UFUNC\_BUFSIZE\_DEFAULT = `10000`

UFUNC\_PYVALS\_NAME = `'UFUNC_PYVALS'`

Uniform = `Uniform (ModelSpec wrapper)`

Vonmisesvariate = `Vonmisesvariate (ModelSpec wrapper)`

WRAP = `1`

Weibullvariate = `Weibullvariate (ModelSpec wrapper)`

Wichmannhill = `Wichmannhill (ModelSpec wrapper)`

absolute = `<ufunc 'absolute'>`

add = `<ufunc 'add'>`

bitwise\_and = `<ufunc 'bitwise_and'>`

bitwise\_not = `<ufunc 'invert'>`

bitwise\_or = `<ufunc 'bitwise_or'>`

bitwise\_xor = `<ufunc 'bitwise_xor'>`

c\_ = `<numpy.lib.index_tricks.CClass object at 0x1154330>`

cast = `{<type 'numpy.int64'>: <function <lambda> at 0x10a8bb0>...`

conj = `<ufunc 'conjugate'>`

conjugate = `<ufunc 'conjugate'>`

copysign = `<ufunc 'copysign'>`

deg2rad = `<ufunc 'deg2rad'>`

divide = `<ufunc 'divide'>`

equal = `<ufunc 'equal'>`

exp2 = `<ufunc 'exp2'>`

expm1 = `<ufunc 'expm1'>`

floor\_divide = `<ufunc 'floor_divide'>`

fmax = `<ufunc 'fmax'>`

fmin = `<ufunc 'fmin'>`

greater\_equal = `<ufunc 'greater_equal'>`

index\_exp = `<numpy.lib.index_tricks.IndexExpression object at ...`

inf = `inf`

infty = `inf`

invert = `<ufunc 'invert'>`

isinf = `<ufunc 'isinf'>`

left\_shift = `<ufunc 'left_shift'>`

little\_endian = `True`

log1p = `<ufunc 'log1p'>`

logaddexp = `<ufunc 'logaddexp'>`

logaddexp2 = `<ufunc 'logaddexp2'>`

logical\_and = `<ufunc 'logical_and'>`

logical\_not = `<ufunc 'logical_not'>`

logical\_xor = `<ufunc 'logical_xor'>`

maximum = `<ufunc 'maximum'>`

mgrid = `<numpy.lib.index_tricks.nd_grid object at 0x113d490>`

minimum = `<ufunc 'minimum'>`

multiply = `<ufunc 'multiply'>`

n = `9`

nan = `nan`

nbytes = `{<type 'numpy.int64'>: 8, <type 'numpy.int16'>: 2, <t...`

negative = `<ufunc 'negative'>`

newaxis = `None`

nextafter = `<ufunc 'nextafter'>`

not\_equal = `<ufunc 'not_equal'>`

ogrid = `<numpy.lib.index_tricks.nd_grid object at 0x113dbb0>`

ones\_like = `<ufunc 'ones_like'>`

r\_ = `<numpy.lib.index_tricks.RClass object at 0x11542f0>`

rad2deg = `<ufunc 'rad2deg'>`

reciprocal = `<ufunc 'reciprocal'>`

remainder = `<ufunc 'remainder'>`

right\_shift = `<ufunc 'right_shift'>`

rint = `<ufunc 'rint'>`

s\_ = `<numpy.lib.index_tricks.IndexExpression object at 0x11543f0>`

sctypeDict = `{0: <type 'numpy.bool_'>, 1: <type 'numpy.int8'>,...`

sctypeNA = `{'?': 'Bool', 'B': 'UInt8', 'Bool': <type 'numpy.bo...`

sctypes = `{'complex': [<type 'numpy.complex64'>, <type 'numpy....`

signbit = `<ufunc 'signbit'>`

spacing = `<ufunc 'spacing'>`

square = `<ufunc 'square'>`

subtract = `<ufunc 'subtract'>`

t = `'0'`

true\_divide = `<ufunc 'true_divide'>`

trunc = `<ufunc 'trunc'>`

typeDict = `{0: <type 'numpy.bool_'>, 1: <type 'numpy.int8'>, 2...`

typeNA = `{'?': 'Bool', 'B': 'UInt8', 'Bool': <type 'numpy.bool...`

typecodes = `{'All': '?bhilqpBHILQPfdgFDGSUVOMm', 'AllFloat': '...`


|  |  |
| --- | --- |
| Variables Details | [hide private] |

### CFGorder

Value:
:   ```
    ['VARSEXT',
     'VARSINT',
     'INPUTS',
     'DEQNS',
     'CFAC',
     'BOUNDS',
     'UNITBOUNDS',
     'GAM1TERM',
    ...
    ```

### nodes\_order

Value:
:   ```
    ['NODE',
     'NODELABEL',
     'NODEADDSTATES',
     'NODESTATEMAP_ACTS',
     'NODESTATEMAP_POTS']
    ```

### ScalarType

Value:
:   ```
    (<type 'int'>,
     <type 'float'>,
     <type 'complex'>,
     <type 'long'>,
     <type 'bool'>,
     <type 'str'>,
     <type 'unicode'>,
     <type 'buffer'>,
    ...
    ```

### cast

Value:
:   ```
    {<type 'numpy.int64'>: <function <lambda> at 0x10a8bb0>, <type 'numpy.
    int16'>: <function <lambda> at 0x10a8bf0>, <type 'numpy.complex128'>: 
    <function <lambda> at 0x10a8c30>, <type 'numpy.uint64'>: <function <la
    mbda> at 0x10a8c70>, <type 'numpy.complex256'>: <function <lambda> at 
    0x10a8cf0>, <type 'numpy.float32'>: <function <lambda> at 0x10a8d30>, 
    <type 'numpy.bool_'>: <function <lambda> at 0x10a8cb0>, <type 'numpy.u
    int8'>: <function <lambda> at 0x10a8db0>, <type 'numpy.int32'>: <funct
    ion <lambda> at 0x10a8eb0>, <type 'numpy.int8'>: <function <lambda> at
    ...
    ```

### index\_exp

Value:
:   ```
    <numpy.lib.index_tricks.IndexExpression object at 0x11543b0>
    ```

### nbytes

Value:
:   ```
    {<type 'numpy.int64'>: 8, <type 'numpy.int16'>: 2, <type 'numpy.comple
    x128'>: 16, <type 'numpy.uint64'>: 8, <type 'numpy.bool_'>: 1, <type '
    numpy.complex256'>: 32, <type 'numpy.float32'>: 4, <type 'numpy.int8'>
    : 1, <type 'numpy.uint8'>: 1, <type 'numpy.uint16'>: 2, <type 'numpy.o
    bject_'>: 4, <type 'numpy.float64'>: 8, <type 'numpy.int32'>: 4, <type
     'numpy.string_'>: 0, <type 'numpy.void'>: 0, <type 'numpy.float128'>:
     16, <type 'numpy.int32'>: 4, <type 'numpy.uint32'>: 4, <type 'numpy.u
    nicode_'>: 0, <type 'numpy.complex64'>: 8, <type 'numpy.uint32'>: 4}
    ```

### sctypeDict

Value:
:   ```
    {0: <type 'numpy.bool_'>,
     1: <type 'numpy.int8'>,
     2: <type 'numpy.uint8'>,
     3: <type 'numpy.int16'>,
     4: <type 'numpy.uint16'>,
     5: <type 'numpy.int32'>,
     6: <type 'numpy.uint32'>,
     7: <type 'numpy.int32'>,
    ...
    ```

### sctypeNA

Value:
:   ```
    {'?': 'Bool',
     'B': 'UInt8',
     'Bool': <type 'numpy.bool_'>,
     'Complex128': <type 'numpy.complex256'>,
     'Complex32': <type 'numpy.complex64'>,
     'Complex64': <type 'numpy.complex128'>,
     'D': 'Complex64',
     'F': 'Complex32',
    ...
    ```

### sctypes

Value:
:   ```
    {'complex': [<type 'numpy.complex64'>,
                 <type 'numpy.complex128'>,
                 <type 'numpy.complex256'>],
     'float': [<type 'numpy.float32'>,
               <type 'numpy.float64'>,
               <type 'numpy.float128'>],
     'int': [<type 'numpy.int8'>,
             <type 'numpy.int16'>,
    ...
    ```

### typeDict

Value:
:   ```
    {0: <type 'numpy.bool_'>,
     1: <type 'numpy.int8'>,
     2: <type 'numpy.uint8'>,
     3: <type 'numpy.int16'>,
     4: <type 'numpy.uint16'>,
     5: <type 'numpy.int32'>,
     6: <type 'numpy.uint32'>,
     7: <type 'numpy.int32'>,
    ...
    ```

### typeNA

Value:
:   ```
    {'?': 'Bool',
     'B': 'UInt8',
     'Bool': <type 'numpy.bool_'>,
     'Complex128': <type 'numpy.complex256'>,
     'Complex32': <type 'numpy.complex64'>,
     'Complex64': <type 'numpy.complex128'>,
     'D': 'Complex64',
     'F': 'Complex32',
    ...
    ```

### typecodes

Value:
:   ```
    {'All': '?bhilqpBHILQPfdgFDGSUVOMm',
     'AllFloat': 'fdgFDG',
     'AllInteger': 'bBhHiIlLqQpP',
     'Character': 'c',
     'Complex': 'FDG',
     'Datetime': 'Mm',
     'Float': 'fdg',
     'Integer': 'bhilqp',
    ...
    ```

  


| Home | Trees | Indices | Help | PyDSTool |
| --- | --- | --- | --- | --- |

|  |  |
| --- | --- |
| Generated by Epydoc 3.0.1 on Fri May 4 15:24:04 2012 | http://epydoc.sourceforge.net |
